# Role of TLR7 expression in cancer: impact on immune activation and prognostic evaluation

**DOI:** 10.1186/s12967-024-05504-0

**Published:** 2024-07-29

**Authors:** Jiafeng Liang, Jie Huang, Yi Tang, Minna Zhang, Lucheng Zhu, Bing Xia

**Affiliations:** Department of Thoracic Oncology, Hangzhou Cancer Hospital, No.34 Yanguan Lane, Shangcheng District, Hangzhou, 310002 China

To the Editor,

Immune therapy is critical to cancer treatment, yet drug resistance remains a formidable challenge [1], underscoring the need for effective treatment predictors. Currently, quantitative assessment of PD-L1 via immunohistochemistry is widely used but exhibits inconsistent predictive capability, prompting exploration of superior biomarkers [2]. Toll-like receptor 7 (TLR7), a member of the Toll-like receptor family, plays a crucial role in pathogen recognition and innate immune activation. Recent studies suggest its involvement in tumor development, with conflicting reports: some indicate TLR7 overexpression promotes tumor growth and drug resistance [3], while others demonstrate that TLR7 agonists inhibit breast, pancreatic, lung, and colorectal cancers through immune activation [4]. Moreover, a PD-L1/TLR7 dual-targeting nanobody-drug conjugate has been developed and shown to exert antitumor effects across multiple tumor models by leveraging innate and adaptive immunity [5]. In the era of immune therapy, further investigation into TLR7’s role and impact in tumor development are crucial.

## Findings

### The expression and prognostic significance of TLR7 across different cancers

**Fig. 1:**
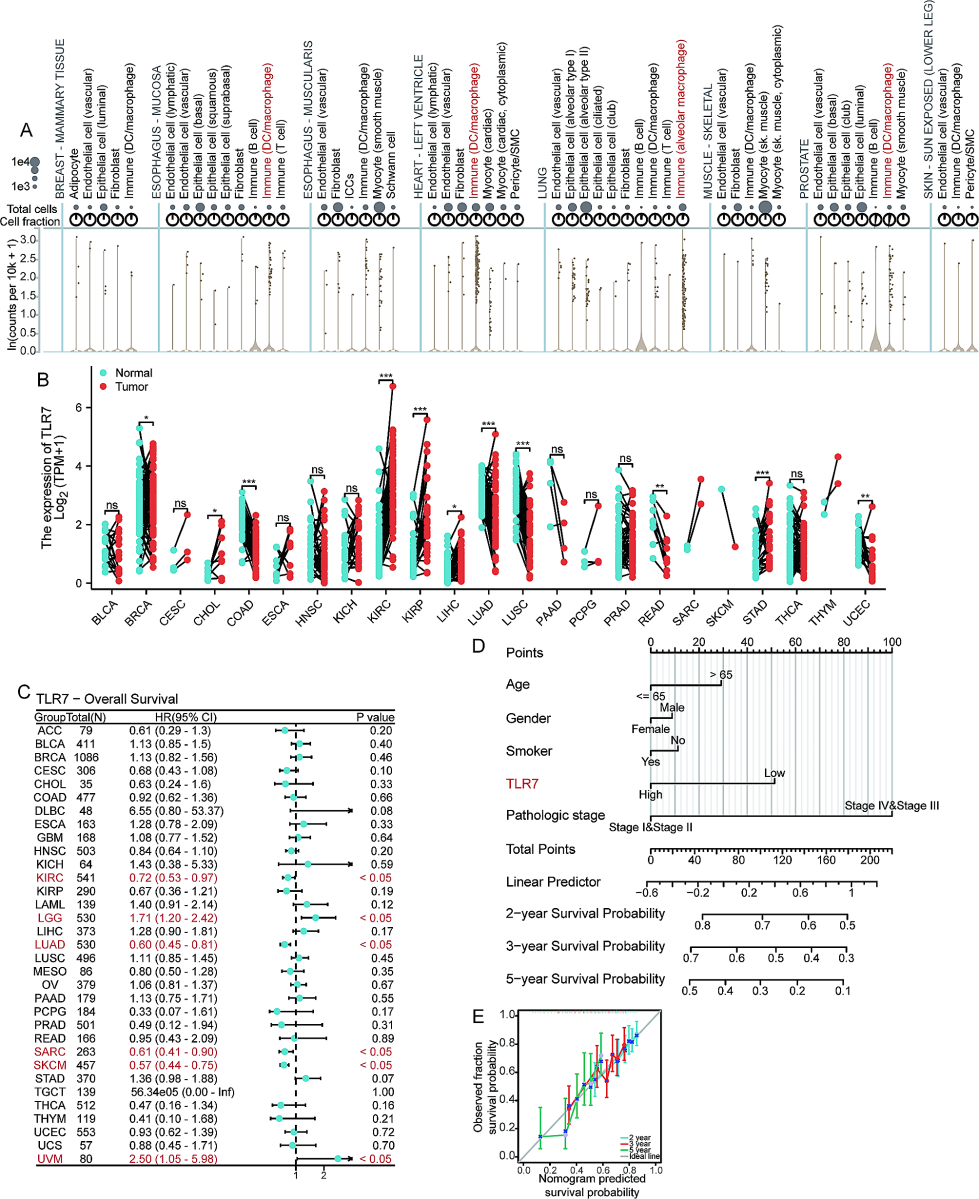
**A**. Complex plots depicted the expression levels of TLR7 among various cell types in different organs. **B**. Paired dot plots illustrated the expression levels of TLR7 in different cancer tissues and corresponding normal tissues. **C**. Forest plot showed the impact of TLR7 expression on overall survival across different cancers. **D**. Nomogram graph demonstrated the significant prognostic role of TLR7 in LUAD. **E**. DCA plot displayed the consistency between nomogram-predicted survival probability and actual survival probability

The distribution of TLR7 in cells was initially explored using single-cell sequencing data from the GTEx portal, revealing predominant expression in macrophages and dendritic cells across various tissues, followed by epithelial cells and other stromal immune cells (Fig. 1A). The biological and pathological roles of TLR7 in cancer were further investigated using data from The Cancer Genome Atlas (TCGA). Comparative analysis of tumor samples with paired normal samples demonstrated reduced TLR7 expression in BLCA, COAD, LUAD, LUSC, READ, and UCEC tumors, while increased expression was observed in CHOL, KIRC, LIHC, and STAD tumors (Fig. 1B). Cox proportional hazards analysis identified TLR7 as a favorable prognostic factor in KIRC, LUAD, SARC, and SKCM, but indicated poorer prognosis in LGG and UVM (Fig. 1C). Furthermore, a nomogram for LUAD constructed an overall survival (OS) prognostic scoring model, validating TLR7 as a significant influencing factor (Fig. 1D). Validation using bootstrapped calibration curves yielded a C-index of 0.650 (95% CI: 0.627–0.673) (Fig. 1E).

### TLR7 serves as an indicator of the tumor immune microenvironment and predicts favorable outcomes with immune checkpoint inhibitors (ICIs) treatment

**Fig. 2:**
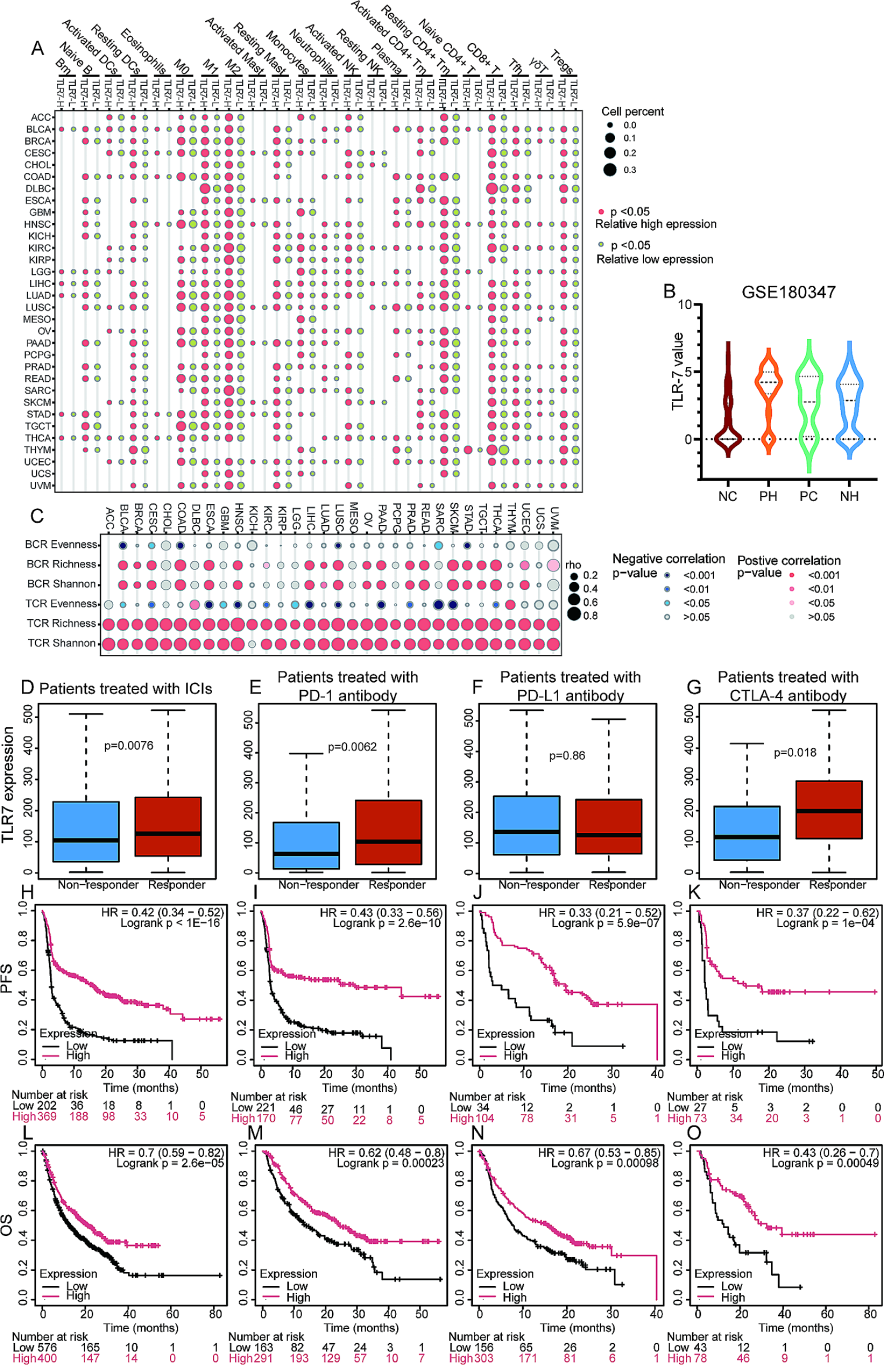
**A**. Dot plots showed the proportions of various immune cells between TLR7-high and TLR7-low groups in different cancers. **B**. Violin plots depicted the expression levels of TLR7 in different microenvironments of LUAD. **C**. Dot plots demonstrated the correlation between immune cell receptor scores and TLR7 expression in different cancers. (**D**-**G**) Box plots displayed the differences in TLR7 expression levels between responders and non-responders to all ICIs (**D**), PD-1 antibody (**E**), PD-L1 antibody (**F**), and CTLA-4 antibody (**G**) treatments. (**H**-**K**) Kaplan–Meier plots showed the PFS differences between TLR7-high and TLR7-low expression groups (grouped by optimal cutoff) under all ICIs (**H**), PD-1 antibody (**I**), PD-L1 antibody (**J**), and CTLA-4 antibody (**K**) treatments. (**L**-**O**) Kaplan–Meier plots displayed the OS differences between TLR7-high and TLR7-low expression groups (grouped by optimal cutoff) under all ICIs (**L**), PD-1 antibody (**M**), PD-L1 antibody (**N**), and CTLA-4 antibody (**O**) treatments. NC, PD-L1 negativity detected by IHC and hematoxylin and eosin showed no immune cell infiltration in the tumor stroma; PH, high PD-L1 expression with immune cell infiltration in the stroma; PC, high PD-L1 expression with no immune cell infiltration in the stroma; NH, low PD-L1 expression with immune cell infiltration in the stroma

As an immune-related molecule, TLR7’s expression in tumors may correlate with immune cell distribution. Cibersortabs analysis consistently showed increases in macrophages, CD8 T cells, resting memory CD4 T cells, resting dendritic cells, and Tregs in TLR7-high expression groups (above or equal to median value). Mast cells, monocytes, neutrophils, activated NK cells, and Tfh cells also generally exhibited elevated levels in TLR7-high groups (Fig. 2A). Overall, TLR7-high expression groups often demonstrated higher immune cell infiltration, whereas TLR7-low expression groups (below median value) exhibited relatively fewer immune cells. Consistent with these findings, LUAD samples stained with hematoxylin and eosin and PD-L1 antibody in the GSE180347 series showed significantly decreased TLR7 expression in PD-L1-negative and “cold” phenotypes (Fig. 2B). Additionally, higher T cell receptor (TCR) richness and Shannon index were observed with elevated TLR7 expression (Fig. 2C), indicating selective TCR amplification in patients with high TLR7 expression. Evidence from the Kaplan–Meier Plotter website supports TLR7 as a tumor immune activator, with responders to ICIs, including PD-1 inhibitors and CTLA-4 inhibitors (Fig. 2D-G), generally exhibiting higher TLR7 expression compared to non-responders. Survival analysis revealed that TLR7-high expression was associated with increased progression-free survival (PFS) and OS benefits across all treatment groups, including PD-1 inhibitors, CTLA-4 inhibitors, and PD-L1 inhibitors (Fig. 2H-O).

## Conclusions

Thus, TLR7, as an immune-related molecule, plays a critical regulatory role in the tumor microenvironment, predicting the prognosis of various cancers. Moreover, high TLR7 expression suggests enhanced immune cell infiltration, particularly CD8 T cells and macrophages, correlating with improved survival with ICIs. Therefore, TLR7 serves as a promising molecular biomarker in immune therapy.

## Data Availability

The source data are available in the TCGA, GEO (GSE18034), GTEX portal (https://gtexportal.org) and Kaplan–Meier Plotter for immunotherapy response evaluation (https://kmplot.com/analysis/index.php?p=service&cancer=immunotherapy). T/B cell receptors (TCR/BCR) data was acquired from Thorsson et al.

## Abbreviations

TLR7: Toll-like receptor 7
TCGA: The Cancer Genome Atlas
OS: Overall survival
PFS: Progression-free survival
ICIs: Immune checkpoint inhibitors
TCR: T cell receptor
